# Physiological and structural traits contribute to thermotolerance in wild Australian cotton species

**DOI:** 10.1093/aob/mcae098

**Published:** 2024-07-09

**Authors:** Garima Dubey, Aaron L Phillips, Darrell J Kemp, Brian J Atwell

**Affiliations:** Hawkesbury Institute for the Environment, University of Western Sydney, Sydney, NSW, Australia; Department of Food Science, University of Adelaide, Adelaide, SA, Australia; School of Natural Sciences, Macquarie University, Sydney, NSW, Australia; School of Natural Sciences, Macquarie University, Sydney, NSW, Australia

**Keywords:** cotton, dissection index, *Gossypium*, heat, leaf shape, leaf surface structures, photosynthesis, relative growth rate, scanning electron microscopy, thermotolerance, trichomes, wild crop relatives

## Abstract

**Background and Aims:**

Five species of cotton (*Gossypium*) were exposed to 38 °C days during early vegetative development. Commercial cotton (*Gossypium hirsutum*) was contrasted with four wild cotton species (*Gossypium australe*, *G. bickii*, *G. robinsonii* and *G. sturtianum*) that are endemic to central and northern Australia.

**Methods:**

Plants were grown at daytime maxima of 30 or 38 °C for 25 days, commencing at the four-leaf stage. Leaf areas and shoot biomass were used to calculate relative rates of growth and specific leaf areas. Leaf gas exchange measurements revealed assimilation and transpiration rates, in addition to electron transport rates and carboxylation efficiency in steady-state conditions. Finally, leaf morphological traits (mean leaf area and leaf shape) were quantified, along with leaf surface decorations, imaged using scanning electron microscopy.

**Key Results:**

Shoot morphology was differentially affected by heat, with three of the four wild species growing faster at 38 than at 30 °C, whereas early growth in *G. hirsutum* was severely inhibited by heat. Areas of individual leaves and the number of leaves both contributed to these contrasting growth responses, with fewer, smaller leaves at 38 °C in *G. hirsutum*. CO_2_ assimilation and transpiration rates of *G. hirsutum* were also dramatically reduced by heat. Cultivated cotton failed to achieve evaporative cooling, contrasting with the transpiration-driven cooling in the wild species. Heat substantially reduced electron transport rates and carboxylation efficiency in *G. hirsutum*, with much smaller effects in the wild species. We speculate that leaf shape, as assessed by invaginations of leaf margins, and leaf size contributed to heat dispersal differentially among the five species. Likewise, reflectance of light radiation was also highly distinctive for each species.

**Conclusions:**

These four wild Australian relatives of cotton have adapted to hot days that are inhibitory to commercial cotton, deploying a range of physiological and structural adaptations to achieve accelerated growth at 38 °C.

## INTRODUCTION

Cotton (genus *Gossypium*), the leading textile fibre plant in the world, is cultivated principally in the sub-tropics between latitudes of 30° and 37° (Singh *et al.*, 2007). Although the primary value of the crop stems from an insatiable world demand for its cellulosic fibre, cotton is also an important source of oil and low-protein livestock feed, thus contributing to global agriculture as a pillar of the economy of many developed and developing countries. Global mean temperature is predicted to increase by ≥3 °C over the next three decades according to climate models, along with more unpredictable and catastrophic weather events, such as intense heatwaves (Calvin *et al.*, 2023). These environmental stresses will inevitably affect the productivity of the world’s agricultural systems, including cotton crops (Hatfield *et al.*, 2011). Although cotton is a warm-climate crop, it is vulnerable to heatwaves at vegetative and reproductive stages (Reddy *et al.*, 1992*a*, *b*; Singh *et al.*, 2007; Masoomi-Aladizgeh *et al.*, 2021). Modelling exercises by Adhikari *et al.* (2016) predicted a 5–17 % reduction in Texan cotton yields by 2070 if temperatures increase as predicted, but with CO_2_ levels held constant in the model; similar reductions in yield are predicted across a wide range of important crop species (e.g. Kuriachen *et al.*, 2022). These impending events have been the impetus for biotechnological approaches to crop improvement, such as genomic manipulations that include incorporation of desired traits to stabilize or enhance yield following a stress event(s). Critically, yield gains through intensification are hoped to mitigate the impact of agriculture on natural ecosystems (Baulcome *et al.*, 2009; Tilman *et al.*, 2011; Crist *et al.*, 2017).

Heatwaves are now recognized to impose an increasing strain on crop production. Cotton crops are commonly irrigated to eliminate drought, whereas heatwaves still pose a palpable threat to productivity. The optimal daytime maximal temperature for cultivation of commercial cotton is ~30 °C (Reddy *et al.*, 1992*b*; Singh *et al.*, 2007; Najeeb *et al.*, 2017; Zafar *et al.*, 2018), whereas temperatures of >36 °C begin to disrupt reproductive processes, such as formation of viable floral structures and gametes (Reddy *et al.*, 1992*a*; Masoomi-Aladizgeh *et al.*, 2021, 2022). As daytime temperatures approach 40 °C, physiological processes in cotton leaves also become impaired (Reddy *et al.*, 1992*c*; Najeeb *et al.*, 2017; Zafar *et al.*, 2018; Masoomi-Aladizgeh *et al.*, 2022). However, the processes that cause this damage are complex, because supra-optimal temperatures simultaneously affect many plant processes, each potentially contributing to growth, development and plant performance (Reddy *et al.*, 1992*c*). For example, sensitivity to heat can be analysed from physiological (e.g. photosynthesis, respiration, solute transport and water relations) and/or structural (e.g. leaf shape, leaf optical properties) perspectives. By experimentation on wild cotton species from arid-zone Australia, we have the opportunity to discover novel traits that have evolved to combat extreme heat regimes in these isolated populations.

Recent interest has turned to wild crop relatives as a source of unique traits and associated alleles that can used for crop improvement programmes (Atwell *et al.*, 2014; Warschefsky *et al.*, 2014; Dempewolf and Guarino, 2015; Dempewolf *et al.*, 2017; Bohra *et al.*, 2022); tolerance to heatwaves has naturally been one focus of this approach (Scafaro *et al.*, 2018). Most crop species have suffered a reduction in genetic diversity owing to intensive and selective crop breeding programmes and domestication, leading to a bottleneck in allelic diversity (Jovovic *et al.*, 2020). The extent to which gene pool diversity has been eroded varies between species, but wild crop relatives are always a rich source of potentially useful genetic diversity (Atwell *et al.*, 2014). Generally, the range of modern cotton crops cannot expand because of existing climatic constraints (Iizumi and Ramankutty, 2015), exacerbated by cycles of climatic extremes, such as heatwaves. Therefore, the germplasm of wild relatives of all crop species endemic to diverse environments is a valuable resource, harbouring a wide range of untapped genetic diversity to tackle abiotic and biotic stress (Vincent *et al.*, 2013; Atwell *et al.*, 2014). In the case of cotton, only four species (*Gossypium hirsutum* L., *G. barbadense* L., *G. arboreum* L. and *G. herbaceum* L.) of ~50 known species are grown commercially. The remaining wild relatives are distributed worldwide, with Mexico having the greatest radiation, followed by Australia (Wendel *et al.*, 2010; Wendel and Grover, 2015). Given their evolutionary history in narrow and harsh ecological niches, these wild relatives are likely to harbour alleles for tolerance to a range of abiotic stresses (Mammadov *et al.*, 2018).

This paper examines leaf physiology and morphology in four of the ~17 Australian wild cotton species in hot daytime conditions. These diploid endemic species, *Gossypium australe*, *G. bickii*, *G. robinsonii* and *G. sturtianum*, represent the G and C genomes of wild *Gossypium*, whereas cultivated cotton, *G. hirsutum*, is an allotetraploid of the A and D genomes (Edwards, 1980; Khan *et al.*, 2000; Brubaker, 2002; Wendel and Grover, 2015). The responses of each of these wild species to a 3-week exposure to 38 °C daytime temperatures were contrasted with a commercial *G. hirsutum* cultivar.

In a series of growth, physiological and anatomical experiments, we tested thee following hypotheses:

1. The natural distribution of wild cotton species throughout the arid zone of Australia indicates selection for thermotolerance traits that are not evident in cultivated cotton.
2. Tolerance to hot days during early vegetative development is manifested in faster shoot relative growth rates in the wild cotton species.
3. Stomatal behaviour and photosynthetic traits also reflect heat tolerance, and leaves of cultivated cotton overheat, leading to impaired photosynthetic processes.
4. Leaf morphology and leaf surface structures have evolved in the wild species to ameliorate the impact of heat.

## MATERIALS AND METHODS

### Plant materials

**Fig. 1.**
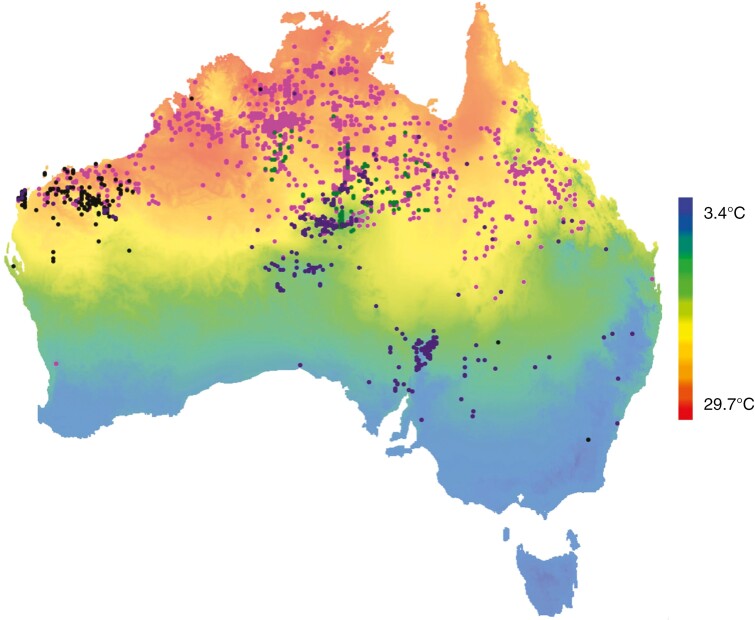
Distribution of four endemic Australian *Gossypium* species (pink, *G. australe*; green, *G. bickii*; purple, *G. sturtianum*; black, *G. robinsonii*). The map also broadly indicates mean annual temperature, with these warm-climate-adapted species concentrated in the northern and north-western regions of the continent with annual mean temperatures >25 °C. This map was generated using the Atlas of Living Australia Interactive Map tool. *Gossypium sturtianum* plants that were sampled in the most southerly and south-easterly parts of the country are unverified as locally adapted populations.

*Gossypium hirsutum* and four Australian wild relatives (*G. australe*, *G. bickii*, *G. robinsonii* and *G. sturtianum*) were grown in temperature-controlled glasshouses at the Plant Growth Facility at Macquarie University (North Ryde, NSW, Australia) from seed sourced from the Australian Grains Genebank with the following accession numbers: *G. australe* (AGG 316844 WCOT); *G. bickii* (AGG 300961 WCOT); *G. robinsonii* (AGG 321721 WCOT); and *G. sturtianum* (AGG 321722 WCOT). A species distribution map with mean annual temperature data overlaid was generated using the interactive map feature of the Atlas of Living Australia (Fig. 1; Belbin, 2011; Belbin *et al.*, 2021). Species were chosen according to their contrasting natural distributions.

### Germination and seedling establishment

Seeds were soaked for 24 h, initially in hot tap water that was left to cool, followed by surface sterilization by soaking in a 2.1 % sodium hypochlorite (White King Premium Bleach) solution for 30 min. They were then rinsed thoroughly with water to remove any traces of bleach and scarified, either by gently abrading with sandpaper or by carefully making nicks in the seed coat with a razor, without damaging the embryo. Five seeds were then placed in multiple closed containers on a pad of moist cotton, covered with moist tissue paper and kept at room temperature in darkness.

Upon emergence of the radicle (3–4 days after imbibition), germinated seeds were transferred into 3-L black polypropylene pots filled with 40:30:30 sand:clay:loam soil. Soil was mixed with 2.5 g L^−1^ of a 70-day slow-release Yates Australia Nutricote N16 Grey fertilizer (N:P:K ratio = 16:4.4:8.3). A fine layer of vermiculite applied to the soil surface was sprayed regularly to maintain soil moisture as seedlings established root systems.

Glasshouse temperatures were set at 30 °C °C/22 °C day/night (12-h photoperiod) for the establishment phase. Natural light was supplemented with blue/red LED light when the light levels fell below 700 µmol photon m^−2^ s^−1^. Established plants were supplied with a commercial water-soluble fertilizer (Aquasol, Yate, Australia; N:P:K ratio = 23:3.95:14) at concentration 1 g L^−1^ every week.

### Growth

For heat treatment, healthy plants at the four- to five-leaf stage were randomly divided into three groups. Four plants from each species were harvested at the commencement of the heat treatment to provide ‘time zero’ weights (i.e. Harvest 1 below) for calculation of growth rates. Leaf areas (LAs) were measured using a LI-3100C Area Meter (Li-Cor, Lincoln, NE, USA). Immediately after area measurements, fresh leaf material was weighed, oven dried at 70 °C for 48 h and reweighed. The remaining plants were maintained in two glasshouses at 30 °C (control) and 38 °C (heat stressed) for 25 days. During heat treatments, plants were watered three times per day (1 min per spray session) using overhead timed spray irrigation to avoid the confounding effects of water deficit and low relative humidity. At the end of the treatment period, a representative plant from each group was photographed. Four healthy and robust plants were harvested after the end of treatments (Harvest 2) for the determination of leaf area and leaf and stem fresh/dry weights (as described above). Relative growth rates (RGRs) of leaf area and biomass were calculated using the formula:


$$\begin{array}{l} RGR=\;\frac{ln\left(area/weight\;Harvest\;2\right)-ln\left(area/weight\;Harvest\;1\right)}{25\;\;days} \end{array}$$

The total number of leaves was recorded at initial and final harvest times. Average individual leaf area was calculated by dividing the total leaf area at final harvest by the total number of leaves at final harvest.

### Dissection index

The perimeter of leaves (*n* = 4) was measured manually using thread. The following formula was applied to obtain a dimensionless measure of the degree of leaf invagination using measured areas of individual leaves for which perimeter had been estimated, with further details available in analysis of vine leaves by Shi *et al.* (2020):


$$\begin{array}{l} Dissection\;index=4\pi A_{L}/{P_{L}}^{2}\; \end{array}$$

Where, *P*_L_ is the perimeter of the leaf and *A*_L_ is the leaf area.

### Gas exchange measurements

Gas exchange variables were measured using a LI-6800 portable photosynthetic system (Li-Cor, Lincoln, NE, USA). The reference chamber was set as follows: flow rate, 400 µmol s^−1^; relative humidity, 40 %; CO_2_, 400 µmol mol^−1^; fan speed, 5000 rpm; light, 1800 µmol m^−2^ s^−1^; and air temperature: 30 or 38 °C, to match the glasshouse temperatures. The youngest healthy, fully expanded leaf from four replicates per species was measured 3 h after the beginning of the photoperiod at each measurement period in the last week of treatment.

### Chlorophyll fluorescence

Chlorophyll fluorescence (of both dark- and light-adapted leaves) was measured using a, LI-6800 with the fluorometer head attached, following the method of Phillips *et al.* (2022). In doing so, the LI-6800 was able to estimate electron transport rates (ETRs).

### Leaf reflectance spectrometry

The ‘Beam method’ of reflectance spectrometry was used to measure leaf reflectance in five cotton species at two temperatures (30 and 38 °C) (Endler, 1990; Kemp and Macedonia, 2006; Kemp, 2008; Badiane *et al.*, 2017). Leaf samples were illuminated at 90° by a pulsed PX-2 xenon light source. An Ocean Optics USB-2000 spectrometer, with the probe angled at 45° to the horizontal surface, was used to capture leaf reflectance. Reflectance was measured as the percentage of light reflected from the leaf surface. To ensure high and equal reflectance in the 300–700 nm spectral range, the spectrometer was calibrated against magnesium oxide white standard after measurement of three replicates of each species, which was done with the standard situated at γ = 0° (i.e. horizontal) (Kemp and Macedonia, 2006; Kemp, 2008).

Four other measures of reflectance were taken at an angle (γ) of 10° rotation from the flat orientation (i.e. rotated towards the observer, away from the observer, clockwise and anticlockwise). Angular deviance was calculated by finding the absolute difference between the mean value for each of these four angled measurements and the ‘flat’ (γ = 0°) value. This value indicates the angle dependence of reflectance from the leaf surface, which provides an estimate of leaf ‘iridescence’.

### Scanning electron microscopy

Three fresh leaves per species were washed with distilled water and cut into ~5 mm pieces. Leaf pieces were dehydrated with a graded series of ethanol: 30, 50, 70, 90, 95 and 2 × 100 % ethanol for 15 min in each ethanol concentration. Samples were dried using a LEICA EM CPD 300 Critical Point Dryer (Leica Microsystems, Wetzlar, Garmany). The dried tissues were mounted on stubs with carbon strips and glue and left overnight for drying. An Emitech K550 gold sputter coater (Quorum Technologies, Lewes, UK) was used for gold-coating the tissues. Finally, tissues were examined with a JEOL JSM-6480LA scanning electron microscope (JEOL, Tokyo, Japan), and images were obtained using JEOL SEM software.

### Statistical analyses

All statistical analyses were performed using GraphPad Prism (v.10.1.0). Species × temperature interaction effects were interrogated using standard two-way ANOVA models. Leaf circularity and reflectance data were analysed using one-way ANOVA models. If the assumptions of heteroscedasticity and/or normality of residuals were not met, the raw data were transformed using the natural logarithm or the square root function. Grubb’s test was used to identify outliers, with these data then excluded from analysis. These infrequent events were ascribed most commonly to the poor performance of an individual plant through mechanical or insect damage to the foliage. For the two-way ANOVAs, all-vs.-all comparisons do not make sense in the context of this study (e.g. it is uninteresting to compare *G. hirsutum* at 30 °C with *G. robinsonii* at 38 °C, etc.); therefore, Sidak’s multiple *post-hoc* comparisons were generated to test the significance of selected groups in a pairwise manner at a significance threshold of 0.05. For the one-way ANOVAs, Tukey’s *post-hoc* comparisons were generated to compare species. All ANOVA tables appear in the Supplementary Data (Tables S1–S15).

## Results

These four endemic *Gossypium* species were collected from savannah and desert sites across northern Australia (Fig. 1). Only *G. sturtianum* has a natural distribution that extends south of the Tropic of Capricorn, comprising two varieties, namely *sturtianum* and *nandewarense*. The former, more widely distributed variety, was used in these experiments. Range areas vary widely among the species, with *G. australe* and *G. sturtianum* endemic to vast areas across northern Australia, whereas *G. bickii* and *G. robinsonii* are endemic to the southern and central regions of the Northern Territory and West Australian Pilbara region, respectively. *Gossypium hirsutum* is an allotetraploid that is believed to have arisen in Central and South America from an ancient hybridization of two wild diploid cotton species; its natural distribution is therefore complex and not represented in Fig. 1.

**Fig. 2.**
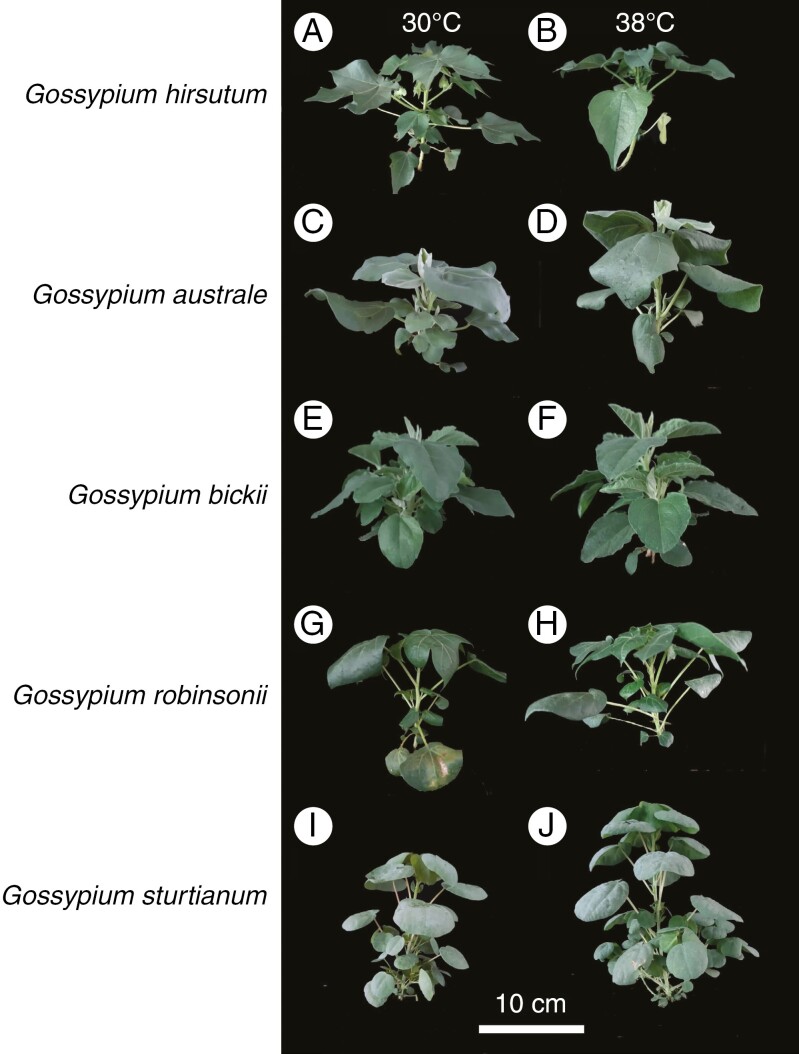
Cultivated and wild *Gossypium* species grown in glasshouses at daytime temperatures of 30 °C (control) and 38 °C (heat) for 3 weeks. Night temperatures were set at 22 °C for all species and both treatments. Scale bar = 10 cm. Images on the left (A, C, E, G, I) show representative plants grown at 30 °C maxima, whereas those on the right (B, D, F, H, J) show plants exposed to 38 °C.

Shoot morphology contrasted strongly among all five species (Fig. 2), with distinctive leaf shapes, numbers and canopy architecture when grown in optimal daytime temperatures (30 °C). Visually, daytime maxima of 38 °C affected the plant height and canopy display of each of the cotton species, enhancing growth in the wild relatives and suppressing growth in *G. hirsutum*. Contrasting effects of heat on the growth and development of each of the five species is corroborated with quantitative data below.

**Fig. 3.**
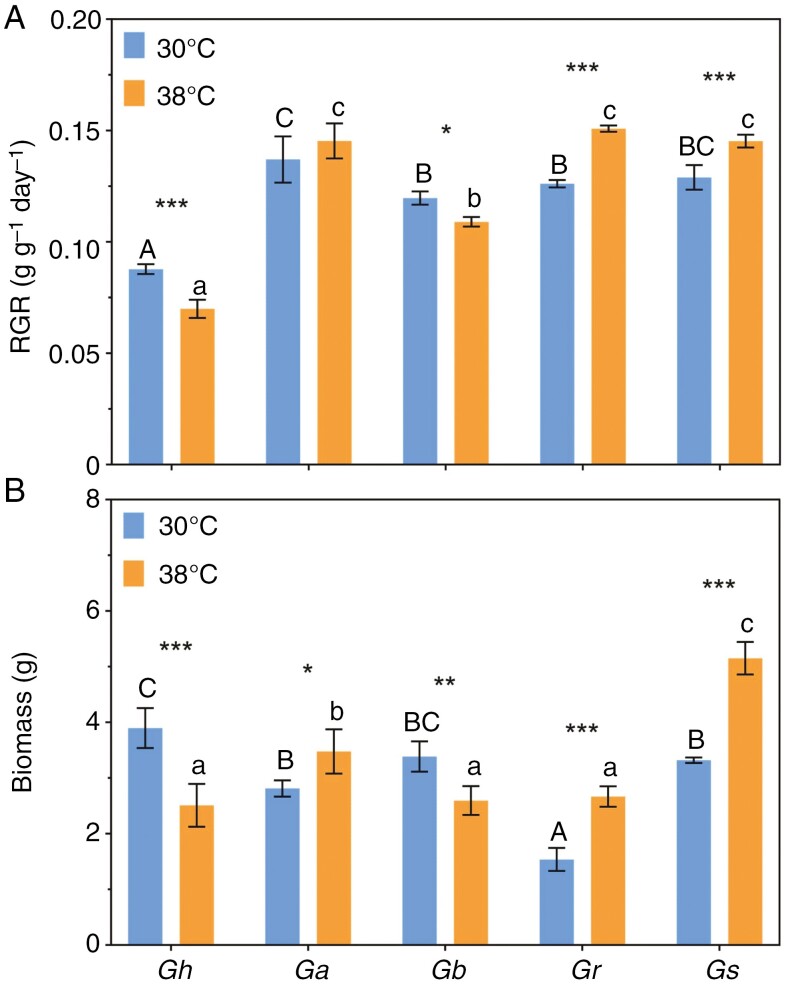
Growth variables of all five *Gossypium* species compared after 25 days at daytime temperatures of 30 or 38 °C. (A) Shoot relative growth rate (RGR). (B) Above-ground dry biomass (leaves plus stem). Bars show means (±s.e., *n* = 4). Asterisks indicate the statistical significance of differences between control and heat-stress treatments within species: *P < 0.05, **P < 0.01 and ***P < 0.001. Different letters (uppercase letters for 30 °C and lowercase for 38 °C) indicate significant grouping information according to *post-hoc* Sidak’s multiple comparison tests.

There was a significant species × temperature interaction for both above-ground biomass (AGB) and RGR (*F*_4,30_ = 45.02, *P*_AGB_ < 0.0001; *F*_4,30_ = 26.11, *P*_RGR_ < 0.0001). Above-ground biomass and relative growth rates of the shoots responded in a similar manner to heat, with *G. sturtianum* and *G. robinsonii* growing faster at 38 than at 30 °C, and *G. hirsutum* plants were significantly smaller after growing for 3 weeks at the higher temperature (Fig. 3). Less definitive responses were observed in *G. australe* (marginally heat tolerant) and *G. bickii* (heat sensitive). Leaf area and the relative rate at which leaf area of the canopy expanded (LARGR) showed the same general trends (*F*_4,30_ = 41.91, *P*_LA_ < 0.0001; *F*_4,39_ = 6.407, *P*_LARGR_ = 0.0005), with *G. hirsutum* negatively affected by 38 °C days (Supplementary Data Figs S1 and S2). Relative growth rates of the four wild species were consistently higher than those of *G. hirsutum* (Fig. 3A), although it should be noted that all plants were the same chronological age and that *G. hirsutum* were the largest plants at 30 °C. The number of leaves followed the same general trends across species and treatments (Supplementary Data Fig. S3A, B), with *G. sturtianum* notable for developing ≤100 leaves in 38 °C heat. Earlier floral initiation in the cultivated cotton could have contributed to a lower RGR. Smaller effects of heat on specific leaf area (SLA) were observed across the species (*F*_4,30_ = 6.939, *P* = 0.0004; Supplementary Data Fig. S4), with the most notable case being *G. hirsutum*, whose leaves had a sharply reduced SLA when grown in 38 °C days; these leaves could therefore be assumed to be thicker and/or denser at 38 °C.

**Fig. 4.**
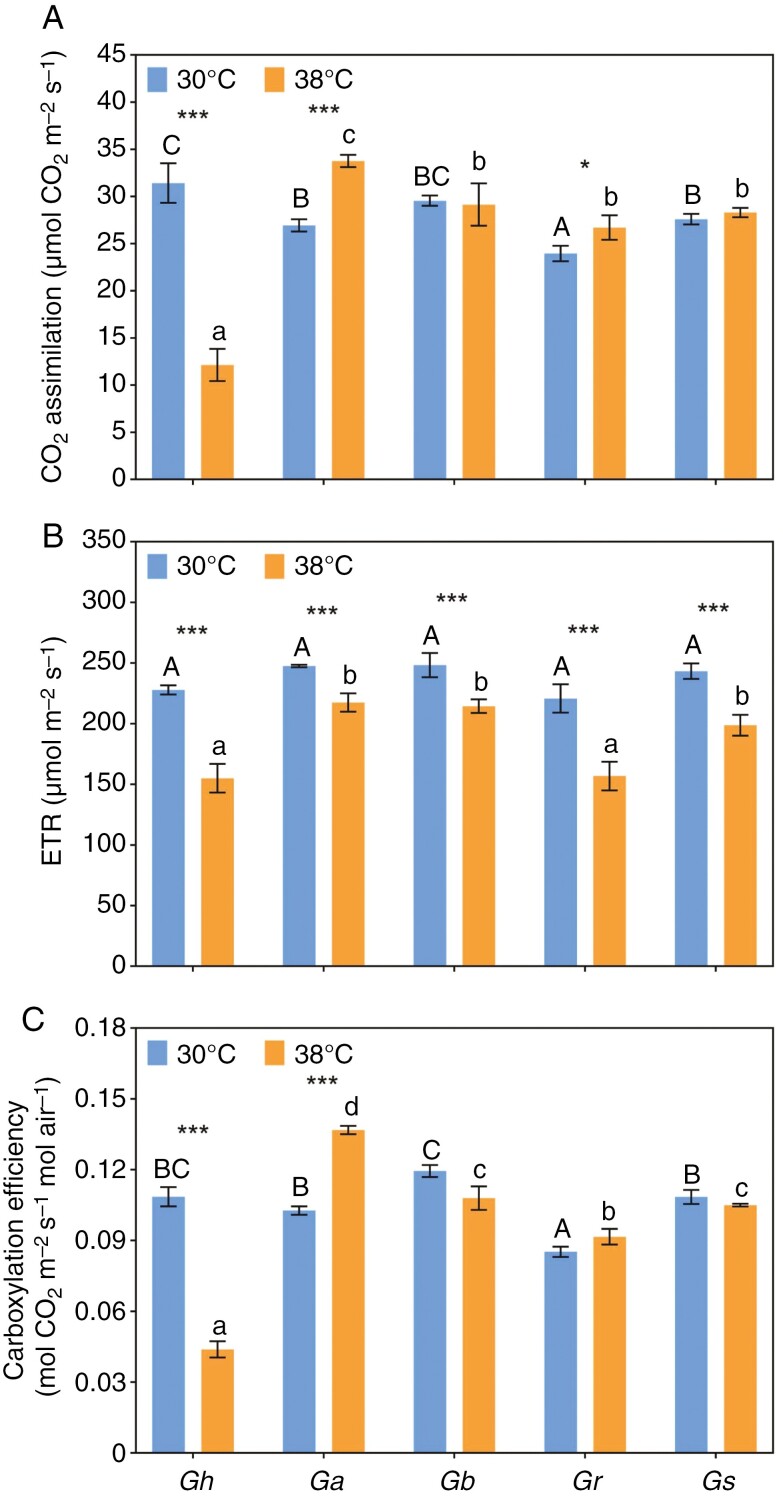
Gas exchange measurements were made on all five *Gossypium* species after 25 days at daytime temperatures of 30 or 38 °C. (A) Photosynthetic rate (*A*). (B) Electron transport rate (ETR). (C) Carboxylation efficiency (CE). Bars show means (±s.e., *n* = 4). Asterisks indicate the statistical significance of differences between control and heat-stress treatments: *P < 0.05, and ***P < 0.001. Different letters (uppercase letters for 30 °C and lowercase for 38 °C) indicate significant grouping information according to *post-hoc* Sidak’s multiple comparison tests.

There were significant species × temperature interactions for both CO_2_ assimilation and CE (*F*_4,30_ = 124.4, *P*_*A*_ < 0.0001 and *F*_4,30_ = 71.80, *P* < 0.0001, respectively), and significant main effects of species and temperature for ETR (*F*_4,30_ = 12.45, *P* < 0.0001 and *F*_4,30_ = 81.69, *P* < 0.0001, respectively). Assimilation rates either increased marginally (*G. australe*) or were unaffected by the 38 °C treatment for the wild species, whereas rates were ~60 % lower when *G. hirsutum* plants were grown at 38 °C (Fig. 4A). Heat reduced CE in *G. hirsutum* by the same proportion (~60 %; Fig. 4C), whereas ETR was only ~33 % lower in *G. hirsutum* plants grown at 38 °C (Fig. 4B). On average, a 15 % reduction in ETR was also observed in the heat-treated wild species. These inhibitory responses to heat were not observed in CE values of the wild species, with *G. australe* carboxylating more efficiently and the other wild species unaffected by 38 °C, whereas CE was strongly inhibited in 38 °C in *G. hirsutum.*

**Fig. 5.**
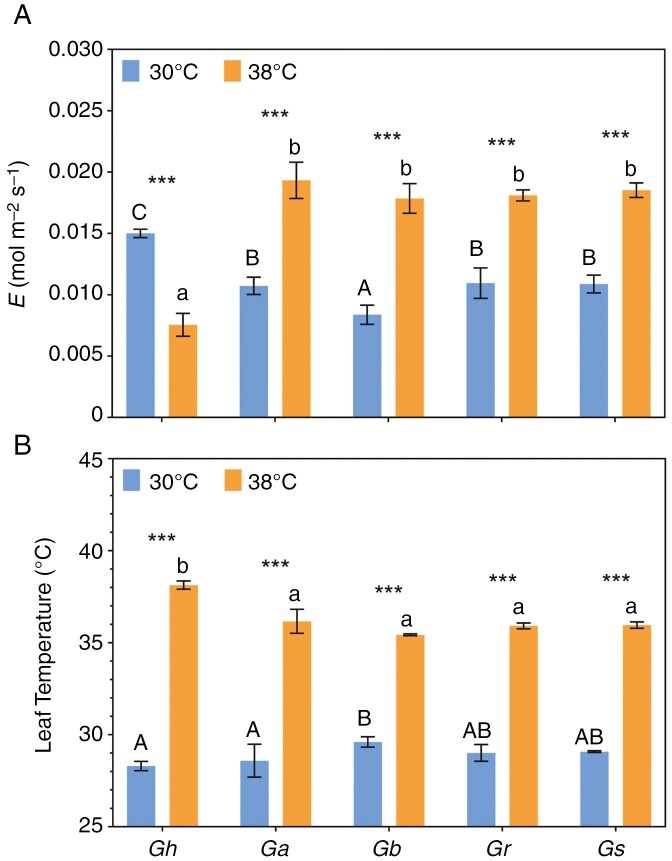
Transpiration (A) and leaf temperature (B) for all five *Gossypium* species after 25 days at daytime temperatures of 30 or 38 °C. Bars show means (±s.e., *n* = 4). Asterisks indicate the statistical significance of differences between control and heat-stress treatments: ***P < 0.001. Different letters (uppercase letters for 30 °C and lowercase for 38 °C) indicate significant grouping information according to *post-hoc* Sidak’s multiple comparison tests.

There was a significant species × temperature interaction for leaf temperature (*F*_4,30_ = 26.48, *P* < 0.0001) and transpiration (*F*_4,30_ = 119.2, *P* < 0.0001). Leaves were predictably hotter in the plants growing at ambient temperatures of 38 °C (Fig. 5B). Greatly reduced transpiration in *G. hirsutum* prevented leaves from cooling, although the wild species were able to cool their leaves by ≤2 °C when grown at 38 °C by significantly upregulating transpiration (Fig. 5A, B).

**Fig. 6.**
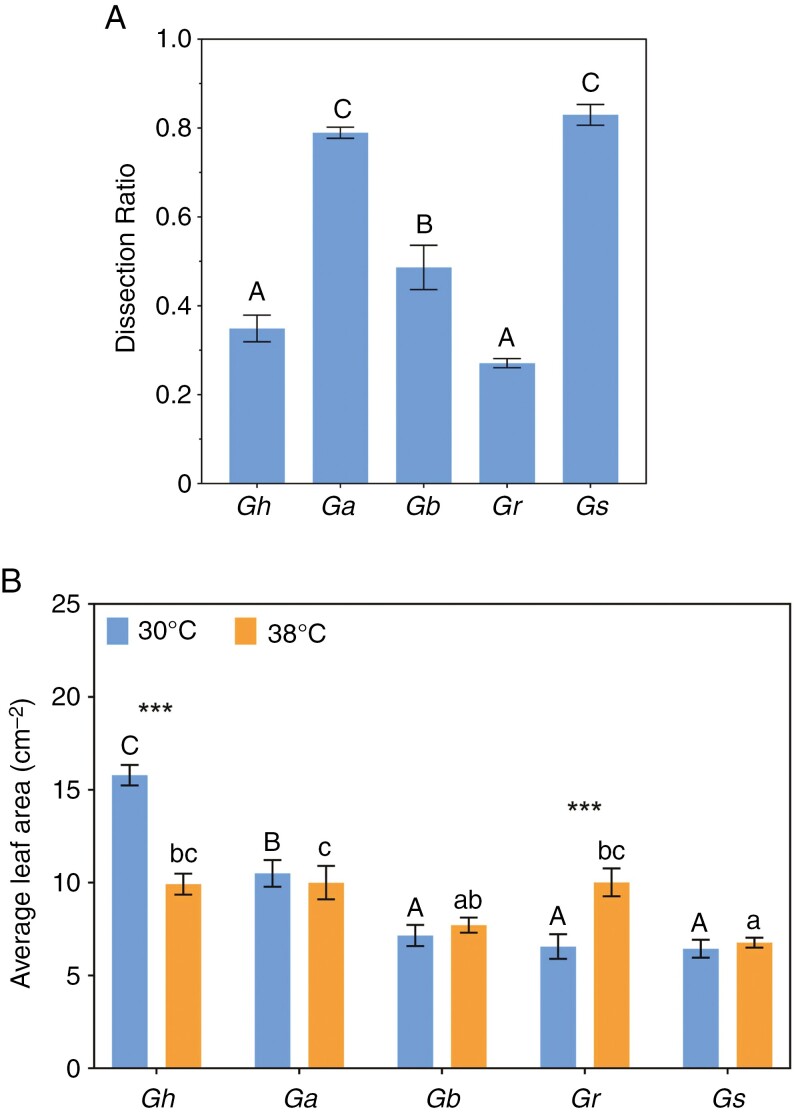
(A) The dissection indices of leaves that grew at daytime maxima of 30 °C. A theoretically highest value (i.e. one) indicates perfect circularity. Means that do not share an uppercase letter are significantly different as determined by a Tukey *post-hoc* test. (B) Average area of individual leaves for plants grown in daytime maxima of 30 or 38 °C. Bars show means (±s.e., *n* = 4). Asterisks in B indicate the statistical significance of differences between control and heat-stress treatments: ***P < 0.001, according to ANOVA results. Different letters (uppercase letters for 30 °C and lowercase for 38 °C) indicate significant grouping information according to *post-hoc* Sidak’s multiple comparison tests.

Leaf invaginations reduce the mean distance from any point in the lamina to the margins; this is a key trait for the dispersal of heat from photosynthesizing leaves (Leigh *et al.*, 2017). Dissection indices are reported for leaves grown in optimal conditions only, but variations in leaf shape were conserved in plants grown at 38 °C. There was a significant effect of species on leaf dissection ratios (*F*_4,15_ = 0.6303, *P* < 0.0001). The increase in leaf perimeter relative to lamina area (low dissection ratio) was greatest for the invaginated leaves of *G. robinsonii* (0.25) and *G. hirsutum* (0.34) and significantly larger for other species, reaching ~0.80 in *G. sturtianum* and *G. australe* (Fig. 6A). Large ‘circular’ leaves naturally have the greatest distance from any mean point on the lamina to the margin, whereas species that have evolved small, invaginated leaves minimize the distance to the leaf margin. There was a significant species × temperature interaction for mean individual leaf areas (*F*_4,39_ = 15.39, *P* < 0.0001). Mean areas of individual leaves were greatly reduced by heat in *G. hirsutum*, whereas *G. robinsonii* leaves were substantially larger when plants were grown at 38 °C (Fig. 6B).

**Fig. 7.**
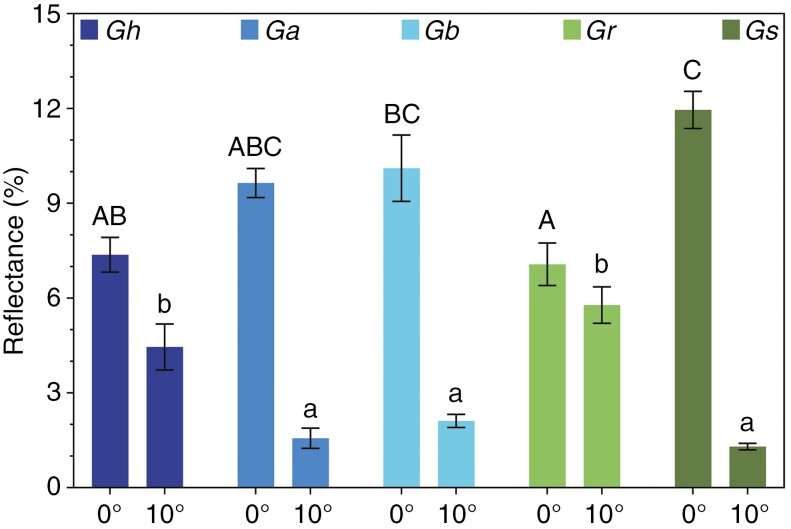
Reflectance (*R*%) of incident light directed onto leaf surfaces of each of the five species after being grown at daytime maxima of 30 °C. Leaves were laid flat, and light was projected onto the surface, either vertically (0°) or at an angle (10°). Different letters (uppercase letters for 0° data and lowercase for 10° data) indicate significant grouping information according to *post-hoc* Tukey’s comparison tests.

**Fig. 8.**
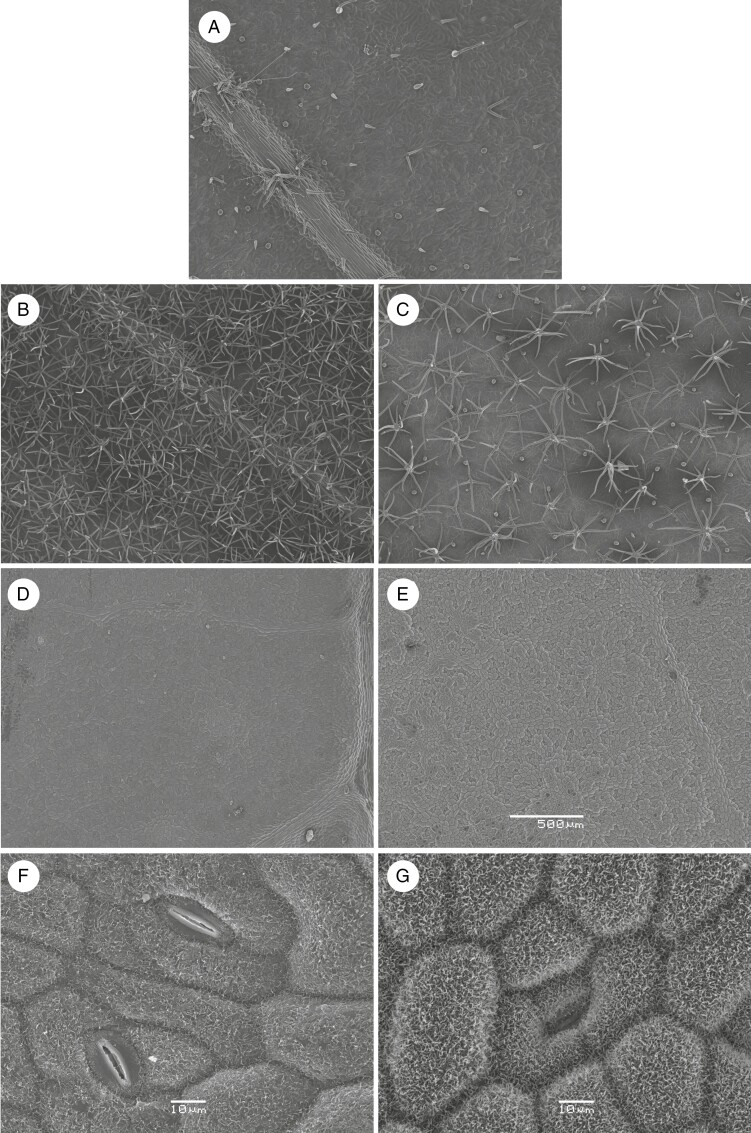
Scanning electron microscopy images of leaf surfaces of five *Gossypium* species: (A) *G. hirsutum*; (B) *G. australe*; (C) *G. bickii*; (D) *G. robinsonii*; and (E) *G. sturtianum*. (F, G) Leaf surfaces of *G. robinsonii* and *G. sturtianum*, respectively, at higher magnification. Plants were grown at daytime maxima of 30 °C. Scale bar: 500 μm in A–E; 10 μm in F, G.

Leaf surface properties were investigated biophysically by directing visible light onto leaf surfaces at two angles (vertical incident light and light at a 10° deviation from the vertical, such as would occur for most of the canopy of non-heliotropic plants during the diurnal cycle). The amount of vertical incident light (0°) that was reflected diverged significantly among the five species (*F*_4,25_ = 0.4587, *P* = 0.0001; Fig. 7). For example, the small, round, silvery leaves of *G. sturtianum* had significantly higher *R*% than leaves of *G. hirsutum* and *G. robinsonii*, with *R*% of the other wild species intermediate. In stark contrast, reflectance of light striking the leaf surface at an angle of 10° was significantly greater for *G. hirsutum* and *G. robinsonii* than that for the other species, presumably relating to the contrasting anatomical features on the leaf surfaces of each species (*F*_4,25_ = 2.008, *P* < 0.0001). We found that leaf surface characteristics were highly diverse (Fig. 8), with large, elaborate and often stellate trichomes on *G. hirsutum*, *G. australe* and *G. bickii*, contrasting with the finer-scale roughened cuticular surfaces on leaves of *G. sturtianum* (Fig. 8D) and *G. robinsonii* (Fig. 8E).

## DISCUSSION

The overarching rationale for this study was that at least one of a selection of four arid-zone wild Australian *Gossypium* species chosen from two genomic clades should have evolved superior thermotolerance not present in a commercially cultivated cotton cultivar (*G. hirsutum*). Although taxonomic relationships have been published for the endemic cotton species of Australia, allocating them to clades and phylogenetic trees (Wendel *et al.*, 2010), there is very little evidence of the functional traits that could reflect the thermotolerance required in their home ranges, and therefore influence the species ranges. This study investigates the sensitivity of early vegetative growth to daytime extreme temperatures that would normally be detrimental to plants, while night temperatures were set at 22 °C in both control and heat-treated glasshouses. Thus, the findings reflect the impact of heat on photosynthesis.

### Natural distribution in relationship to temperature

The four *Gossypium* species in this study have distinct natural distributions throughout the Australian savannah and northern tropics (Fig. 1), whereas the commercial cotton cultivar (*G. hirsutum*) is cultivated mainly in irrigation systems in south-eastern Australia. The endemic species used in this study are a subset of what are believed to be 17 native *Gossypium* species (Wendel *et al.*, 2010; Wendel and Grover, 2015; Mammadov *et al.*, 2018) and do not include the K genome species from the north-west of the country. However, they represent two major genomes, namely the C genome (*G. sturtianum* and *G. robinsonii*) and the G genome (*G. bickii* and *G. australe*), although *G. australe* is clustered with the C genome species in a DNA fingerprinting study (Khan *et al.*, 2000). Significantly, the relatively narrow natural distributions of *G. robinsonii* and *G. bickii*, found in the Pilbara of Western Australia and Central Australia, respectively, coincide with the hottest landscapes in the continent. The other two wild species are far more widely distributed, occurring naturally across the northern savannah of Australia. Climate and soil factors are both likely to contribute to these divergent home ranges.

### Growth and development: species × temperature interactions

*Gossypium hirsutum* shoots were visibly smaller after 25 days of heat treatment when compared with the four *Gossypium* wild relatives, which appeared to benefit from the higher temperature (Fig. 2).

Lower RGR after 25 days of heat treatment resulted in a 40 % reduction in shoot biomass of *G*. *hirsutum* (Fig. 3). In heat-stress experiments on Pima cotton (*Gossypium barbadense*), Reddy *et al.* (1992*c*) reported an optimal temperature regime of 30 °C/22 °C (day/night) for maximal mainstem elongation, leaf area growth rate and biomass accumulation, with growth rates on a leaf area basis reduced by 50 % when plants were exposed to 40 °C (relative to 30 °C). This reduction in growth in Pima cotton stood in sharp contrast to the wild cotton species in our experiment, in which 38 °C enhanced growth in three species, with only a slight reduction in *G. bickii*. Notably, it was the two C-genome species (*G. robinsonii* and *G. sturtianum*) that were 50–70 % heavier after growing at 38 °C. Because RGR is derived from the exponential phase of growth in this study, numerically small differences in RGR translated to major impacts on biomass after only 25 days of heat (Fig. 3B).

Variation in growth rates through heat treatment was the result of highly species-specific differences in both leaf size (Fig. 6B) and the number of leaves (Supplementary Data Fig. S3A, B). For example, the C-genome species had either larger leaves when subjected to heat (*G. robinsonii*) or more leaves (*G. sturtianum*), culminating in larger overall leaf area in these species, whereas *G. hirsutum* had both fewer and smaller leaves (Fig. 6; Supplementary Data Fig. S1). Heat had no effect on leaf area in *G. bickii* and *G. australe*. The contrasting effects of heat on the area of the leaf canopy in each species were reflected in the relative rate of leaf area increase, showing that expansion of leaf tissues in the C-genome species was relatively faster at 38 °C, whereas the laminae of *G. hirsutum* expanded more slowly during heat treatment (Supplementary Data Fig. S2). Similar reductions in individual leaf size and numbers of leaves (i.e. rate of leaf appearance) have been reported in drought conditions (Pettigrew, 2004; Fahad *et al.*, 2017), but there appear to be no consistent patterns for plants subjected to heat. This study shows that even in closely related wild species, leaf initiation (development) and leaf expansion (growth) respond very differently during exposure to high daytime temperatures.

The disjunct between biomass and area of leaves is captured in the SLA (Supplementary Data Fig. S4), an important leaf trait that has strong plasticity in response to many environmental and genetic factors (Wright *et al.*, 2004; Zhou *et al.*, 2020). Californian species growing in desert conditions had smaller SLA (thicker or denser leaves) with more resilient photosynthetic electron transport systems than coastal species with greater SLA (Knight and Ackerly, 2003). Accordingly, the authors claimed that lower SLA and smaller leaf size are typical of species growing in hot and arid regions, arising through a series of convergent evolutionary events. Individual leaves of *G. hirsutum* were on average twice the area of the wild relatives when grown at 30 °C (Fig. 6B), consistent with its intense selection for photosynthetic performance in well-watered field conditions (Fonseca *et al.*, 2000). When daytime temperatures were set at 38 °C, SLA of *G. hirsutum* decreased strongly, indicating a capacity to mount a response to higher air temperatures. A weaker decrease in SLA was seen in *G. robinsonii* when grown at 38 °C, consistent with the suggestion by Nicotra *et al.* (2008) that SLA responds interactively with leaf dissection in hot conditions, whereas SLA was not affected by heat in the other three naturally heat-adapted wild relatives. Our data indicate that wild cotton relatives appear to have evolved relatively high SLAs as a genetically fixed trait that does not respond consistently to the heat typical of their natural ranges.

### Heat has a differential effect on photosynthetic performance

We tested the hypothesis that photosynthetic performance in one or more of the wild cotton relatives is more resilient to heat than observed in the cultivated species. Because genetic differences in thermotolerance were largely expressed before leaf canopies commenced self-shading (i.e. in young, expanding leaf canopies), we speculate that thermotolerance was related to direct effects of heat on photosynthetic efficiency in these fully illuminated canopies. It is important to emphasize that plants were well watered throughout the experiment in humid glasshouses; therefore, they were never subject to water deficits.

The CO_2_ assimilation rate (*A*) of *G. hirsutum* declined by ~60 % at 38 °C (Fig. 4A), supporting the findings of Feller *et al.* (1998) and Law and Crafts-Brandner (1999), who showed a substantial decrease in light-saturated CO_2_ exchange at 38 °C in a *G. hirsutum* cultivar. Carboxylation efficiency (CE), derived from the initial slope of the *A*/*C*_i_ (*A*, net CO_2_ assimilation rate; *C_i_*, versus calculated substomatal CO_2_ concentration) curve, reflects the efficiency of CO_2_ fixation in the Calvin cycle and has been shown to be impaired by heat-induced damage to the catalytic efficiency of Rubisco, even at 35 °C (Feller *et al.*, 1998; Scafaro *et al.*, 2016). Notwithstanding the heat tolerance generally ascribed to commercial cotton varieties (Carmo-Silva and Salvucci, 2011), the dramatic inhibition of CE at 38 °C in *G. hirsutum* contrasted with non-significant or enhanced CE in the wild relatives at the higher temperature (Fig. 4C). Various mechanisms could be invoked to explain this difference, including a dysfunctional interaction between Rubisco activase and Rubisco (Crafts-Brandner and Salvucci, 2000). There is prior evidence of species within a single genus having natural variation in heat tolerance as a result of the thermostability of Rubisco activase, with an endemic wild Australian rice species having a more thermally stable Rubisco activase than domestic rice at 40 °C (Scafaro *et al.*, 2016, 2018). At high temperatures, the specificity of Rubisco for CO_2_ (compared with O_2_) declines, increasing rates of photorespiration and lowering CE (Walker *et al.*, 2016). The geographical ranges of the four wild cotton species would strongly suggest that traits that reduce photorespiration might have evolved, notwithstanding that these are C_3_ species without a CO_2_-concentrating mechanism (Gowik and Westhoff, 2011; Langdale, 2011; Guidi *et al.*, 2019). According to Perry *et al.* (1983), photorespiration represented ~50 % of net photosynthesis in cotton when the air temperature was 40 °C, thus amelioration of this burden on photosynthetic performance would be under high selective pressure in arid-zone species. In short, the absence of any negative impact of 38 °C on photosynthetic rate and CE of *G. bickii*, *G. robinsonii* and *G. sturtianum* makes a strong case for thermotolerance mechanisms having evolved in the wild, while *G. australe* had significantly faster *A* and higher CE when grown for several weeks at 38 °C (Fig. 4C).

Alternative explanations for the relative sensitivity of *G. hirsutum* to heat include damage to photochemical processes, manifested as reduced quantum yield (Bibi *et al.*, 2008; Guidi *et al.*, 2019) and limitation of triose phosphate utilization (McClain and Sharkey, 2019). Heat-induced injury to the coupling of photosynthetic electron transport to energy transductions was evident in all five species under investigation (Fig. 4B). For example, there were substantial effects of 38 °C days on ETR, with reductions ≤30 % in *G. hirsutum* and the wild relative, *G. robinsonii* (Fig. 4B). The substantial impact of heat on photochemistry in all five cotton species tested translated to reduced assimilation rates only in *G. hirsutum*, suggesting that CE, which was also reduced only in *G. hirsutum* in hot conditions, was the key determinant of thermotolerance in cotton.

High stomatal conductance rates in smaller leaves increase evaporative water loss and reduce heat loads, as demonstrated in Pima cotton (Lu *et al.*, 1994, 1997; Radin *et al.*, 1994). Stomatal conductance declined by ~80 % when *G. hirsutum* grew at 38 °C (Supplementary Data Fig. S5), causing the transpiration rate to decrease by 50 %, even when plants were supplied continuously with water (Fig. 5A). In contrast, in the wild species, stomatal conductance was either steady or significantly higher in the heat-treated plants, with transpiration significantly greater at 38 °C (Fig. 5A). The contrasting effects on leaf cooling were therefore stark, with *G. hirsutum* unable to exert any leaf cooling via transpiration, whereas the wild relatives cooled their leaves effectively (Fig. 5B). Overheating of leaves would have had an immediate impact on metabolism, thereby explaining the differential impact on *G. hirsutum* compared with the wild relatives. For example, a plot of leaf temperature vs. assimilation rates illustrates the distinctively negative impact of heat in *G. hirsutum* compared with the wild relatives (Supplementary Fig. S6). In a related study, Deva *et al.* (2020) reported that heat-tolerant genotypes of common bean cooled their leaves more than heat-sensitive genotypes because of enhanced transpirational cooling caused by increased stomatal conductance. A case can be made for more focus on transpirational cooling as a favourable trait, in part relieving the need for metabolic modifications aimed at improved thermotolerance. Small temperature differences can have profound effects on biochemical processes in leaves and therefore downstream impacts on plant performance and growth rates (Scafaro and Atkin, 2016). The relationship between assimilation and relative growth rates illustrates this in the present study, where the four wild species photosynthesized and grew faster at 38 °C, whereas these processes were impaired in the cultivated species (Supplementary Fig. S7).

### Structural traits contribute to thermotolerance

In addition to leaf cooling by transpiration, surface structures on leaves can make a major contribution to thermotolerance through increasing reflectance (Ehleringer *et al.*, 1976) and enhanced dispersal of sensible heat (Lin *et al.*, 2017). Pubescence promotes scattering and reflectance of excess light at high temperatures, reducing heat loads, transpirational water loss and damage from excess light. Species endemic to hot and dry conditions have often evolved structures to ameliorate excessive heat loads by reflecting extra radiation; these can be in the form of wax or trichomes on leaf surfaces (Ehleringer and Björkman, 1978; Ehleringer, 1982; Curtis *et al.*, 2012). Ehleringer *et al.* (1976) reported that the pubescent layer in *Encelia farinosa* acts as blanket reflector because it reduces leaf heat load by reflecting 70 % and absorbing only 29 % of solar radiation in the visible range, resulting in reduced leaf temperature. In this study, we observed stark differences in leaf surface architectures between the five *Gossypium* species (Fig. 8). We speculate that the presence of stellate trichomes on the surfaces of *G. australe* and *G. bickii* (Fig. 8B, C) is more closely related to drought tolerance, whereas the ‘fuzzy’ texture of leaf surfaces of *G. robinsonii* and *G. sturtianum* (Fig. 8D–G) that can be seen at high magnification evolved to reflect radiant heat. The sharp contrasts in surface structures are most apparent when the same scale is used to compare each of the species (Supplementary Fig. S8). The reflectance of *G. hirsutum* leaves in the visible spectrum was at the lower end of the range, suggesting that its leaves were more likely to be damaged by excess energy (Fig. 7). The C-genome species had surprising optical properties: the highest reflectance and lowest angular deviance in *G. sturtianum* in the visible range suggested a ‘shiny’ surface coated with a smooth waxy cuticle as one strategy to reduce heat gain, whereas *G. robinsonii* was equally effective at reflecting light whether the radiation was perpendicular to the leaf surface or directed at an angle of 10°. Although the precise mechanisms of heat dispersal that result from these leaf surface structures requires more detailed analysis, the prediction that the leaves of the wild species have evolved structures that can reflect radiant energy from underlying tissues was generally upheld. This conclusion is consistent with significantly lower leaf temperatures of the wild relatives when growing at 38 °C when compared with *G. hirsutum*. Inconclusive findings on the effectiveness with which these species reflected infrared radiation (data not shown) and the impact of high temperatures on development of these varies leaf surface structures remain to be elucidated further.

Variability in leaf area and shape plays a vital role in leaf thermal regulation through its influence on leaf boundary layer thickness (Leigh *et al.*, 2017). Yates *et al.* (2010) reported a positive correlation between leaf temperature and leaf size in a range of South African Proteaceae. Environments that are hot, sunny and arid, such as those in which the four wild cotton relatives in our study have evolved, typically support small-leaved species (Wright *et al.*, 2017). Curiously, leaves of *G. hirsutum*, although being the largest of all five species in optimal conditions, were significantly smaller at 38 °C (Fig. 6B), either as part of an adaptive response to heat or as a result of impaired expansion growth. In contrast, leaves of *G. robinsonii* were 50 % larger at 38 °C (Fig. 6B), notwithstanding the high degree of thermotolerance in that species. Clearly, multiple factors beyond leaf size influence heat tolerance in these species (e.g. leaf shape).

Although leaf shape (distance to leaf margins) affects heat tolerance, it probably did not evolve directly or uniformly as a mechanism for thermal regulation (Nicotra *et al.*, 2011; Leigh *et al.*, 2017). Invaginations (‘dissection’) of leaf margins contribute to loss of sensible heat by reducing the distance from every point in the lamina to the leaf margin (Leigh *et al.*, 2017). Variation in leaf shape from simple to complex laminae (‘toothed’ or ‘lobed’) has been associated with increased photosynthesis and transpiration in early stages of growth in cold-climate species (Royer and Wilf, 2006); our findings in wild cotton suggest a related phenomenon in *G. robinsonii* during heat. Using a dissection index that ranges up to one for a perfectly circular leaf and less as leaf margins become invaginated, the distinctively lobed leaves of *G. robinsonii* and *G. hirsutum* appear to have a near-ideal shape for high photosynthetic rates in optimal growing conditions (for *G. hirsutum*) and high temperatures in their natural range (for *G. robinsonii*) through heat dispersal.

Our results suggest that in general, the wild species had smaller leaves than *G. hirsutum*, whether they were lobed or not (Fig. 6A, B). For example, *G. sturtianum* had almost perfectly circular, small leaves, with increasing levels of invagination in *G. australe*, *G. bickii* and *G. robinsonii* (Fig. 6A, B). It appears that leaf size and shape are strongly genetically determined, interacting traits, each acting to acclimatize to overheating in their natural environments. Furthermore, leaf size responded to heat, indicating a degree of phenotypic plasticity in leaf development, although impairment to rates of expansion of leaf tissues at high temperatures as they emerged from the apical meristems might have played a role in the ultimate leaf areas of *G. hirsutum*.

## Conclusions

Four wild Australian cotton species had varying degrees of tolerance to high daytime temperatures, and all species were more heat tolerant than cultivated cotton. For example, growth in both C-genome species (*G. robinsonii* and *G. sturtianum*) was generally more tolerant to heat than the G-genome species (*G. bickii* and *G. australe*). Photosynthetic rates and associated electron transport and carboxylation efficiency were strongly inhibited in *G. hirsutum* compared with the wild relatives. However, even within the C-genome species, there were contrasting responses to heat. For example, *G. sturtianum* produced many near-circular leaves, increasing leaf production in hot conditions, whereas its C-genome co-species, *G. robinsonii*, had lobed leaves that were larger in hot conditions without a substantial increase in the number of leaves. Despite the generally similar hot, high-radiation and arid conditions in which all four wild cotton species evolved, a combination of physiological (e.g. CO_2_ assimilation) and structural (e.g. leaf morphology, surface ultrastructure) features contributed to survival in these harsh conditions. Commercial cotton, in contrast, was relatively sensitive to days at 38 °C, despite its general reputation as a heat-tolerant crop species.

## SUPPLEMENTARY DATA

Supplementary data are available at *Annals of Botany* online and consist of the following.

Figure S1: total leaf area per plant. Figure S2: relative rate of growth in leaf area. Figure S3: number of leaves for plants at 30 and 38 °C. Figure S4: specific leaf area. Figure S5: stomatal conductance. Figure S6: assimilation vs. leaf temperature (symbols: closed = 30 °C; open = 38 °C). Figure S7: relative growth rate vs. assimilation rate (symbols: closed = 30 °C; open = 38 °C). Figure S8: Scanning electron micrographs of leaf surfaces, each at the same magnification (see 10 µm scale bar). Table S1: ANOVA table for relative growth rate (RGR). Table S2: ANOVA table for above ground biomass. Table S3: ANOVA table for CO_2_ assimilation. Table S4: ANOVA table for electron transport rate (ETR). Table S5: ANOVA table for carboxylation efficiency. Table S6: ANOVA table for transpiration (*E*). Table S7: ANOVA table for leaf temperature. Table S8: ANOVA table for leaf dissection ratio. Table S9: ANOVA table for average individual leaf area. Table S10: ANOVA table for reflectance at 0°. Table S11: ANOVA table for reflectance at 10°. Table S12: ANOVA table for total leaf area. Table S13: ANOVA table for leaf area relative growth rate (RGR). Table S14: ANOVA table for specific leaf area (SLA). Table S15: ANOVA table for stomatal conductance to water (*g*_*sw*_).

mcae098_suppl_Supplementary_Figures

mcae098_suppl_Supplementary_Tables_S1-S15

## Funding

This project was the result of postgraduate student projects (G.D. and A.L.P.) and received no external or internal funding beyond partial Faculty support provided for graduate studies within Macquarie University.
