# Supplementary figures and images for: Physiological and structural traits contribute to thermotolerance in wild Australian cotton species
Source: Ann Bot. 2024 Jul 9;135(3):577–88. doi: 10.1093/aob/mcae098 (PMC11897598; doi:10.1093/aob/mcae098)

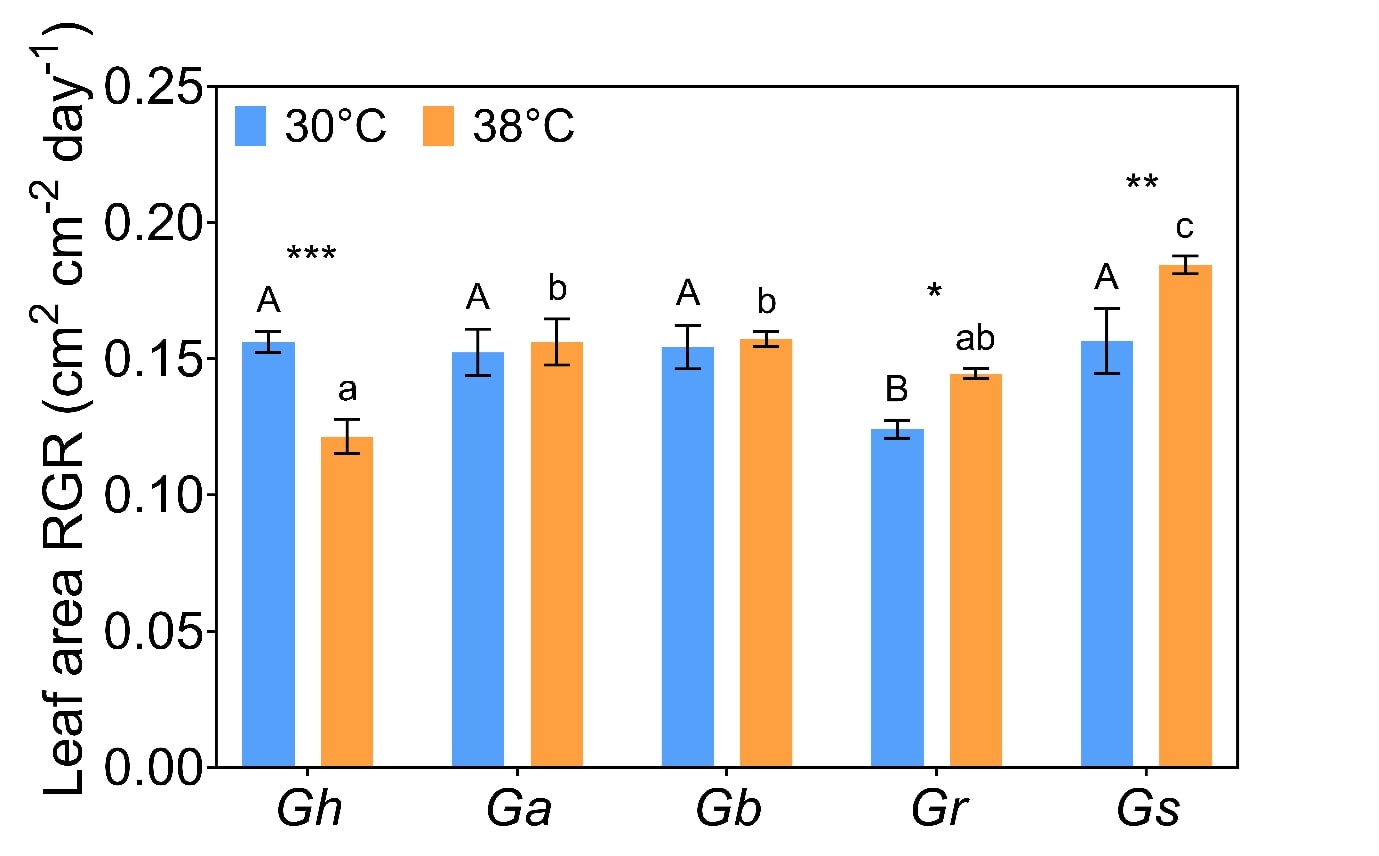

Supplement: mcae098_suppl_Supplementary_Figures [file mcae098_suppl_supplementary_figures.zip › aob-24221-s03.jpg]

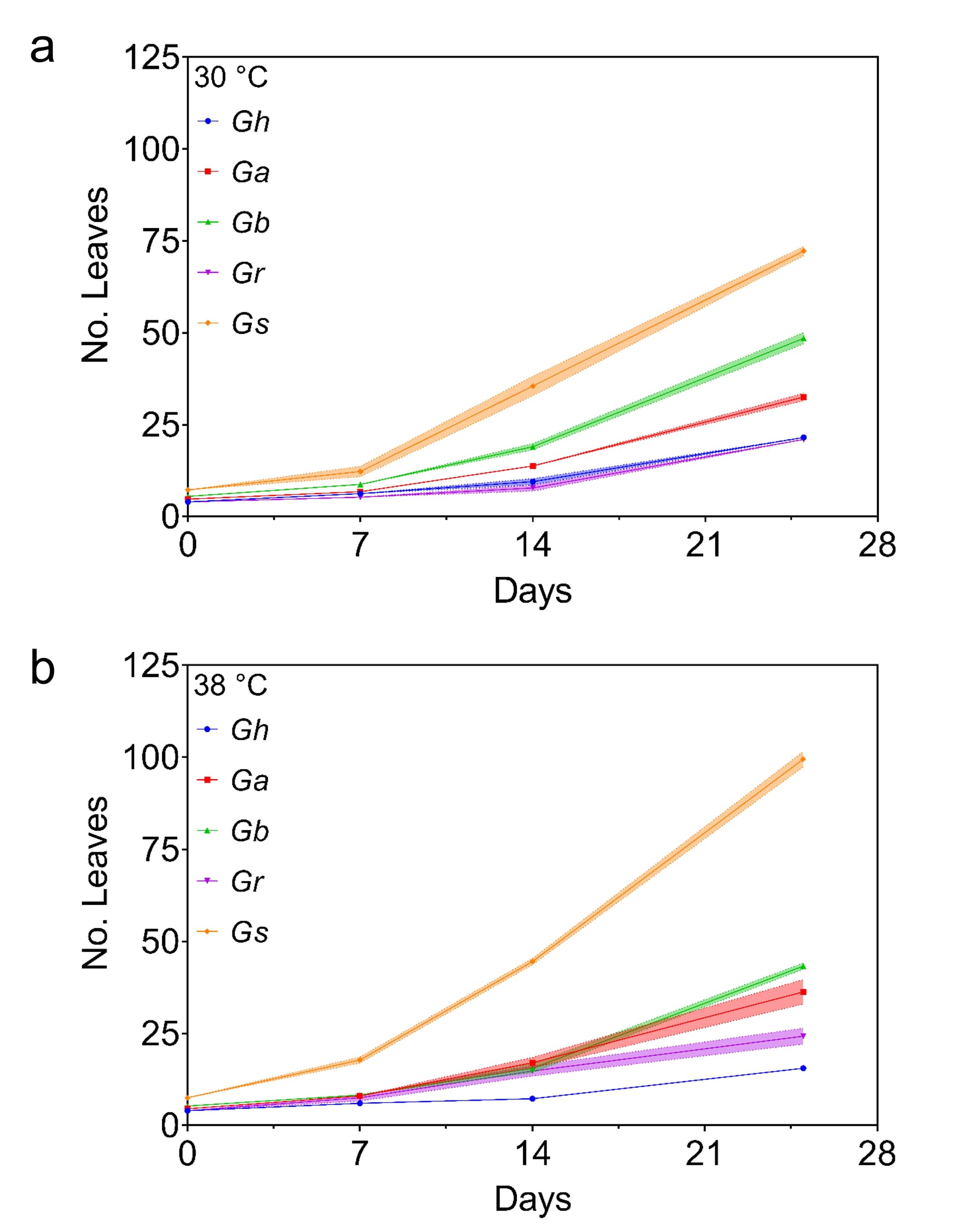

Supplement: mcae098_suppl_Supplementary_Figures [file mcae098_suppl_supplementary_figures.zip › aob-24221-s04.jpg]

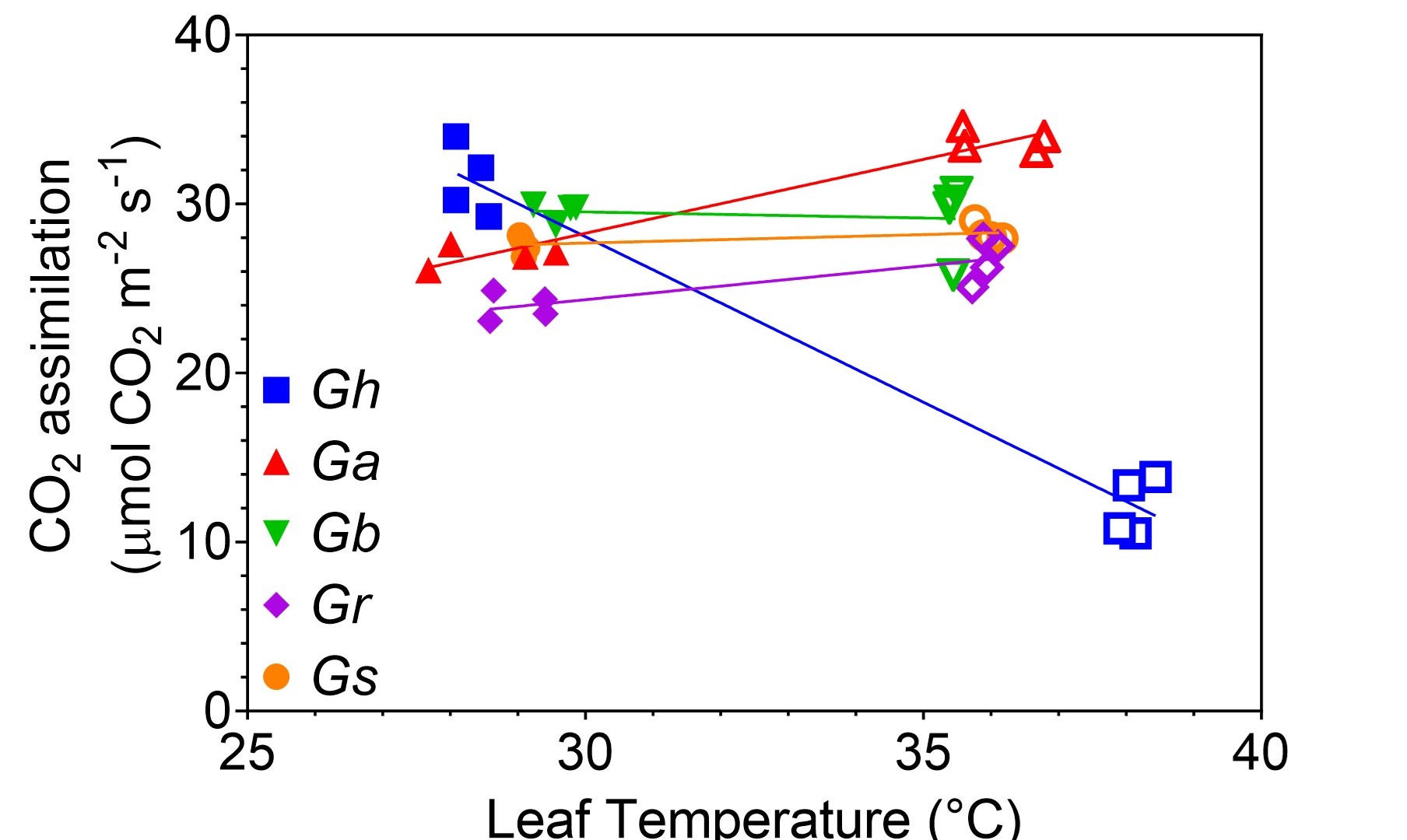

Supplement: mcae098_suppl_Supplementary_Figures [file mcae098_suppl_supplementary_figures.zip › aob-24221-s07.jpg]

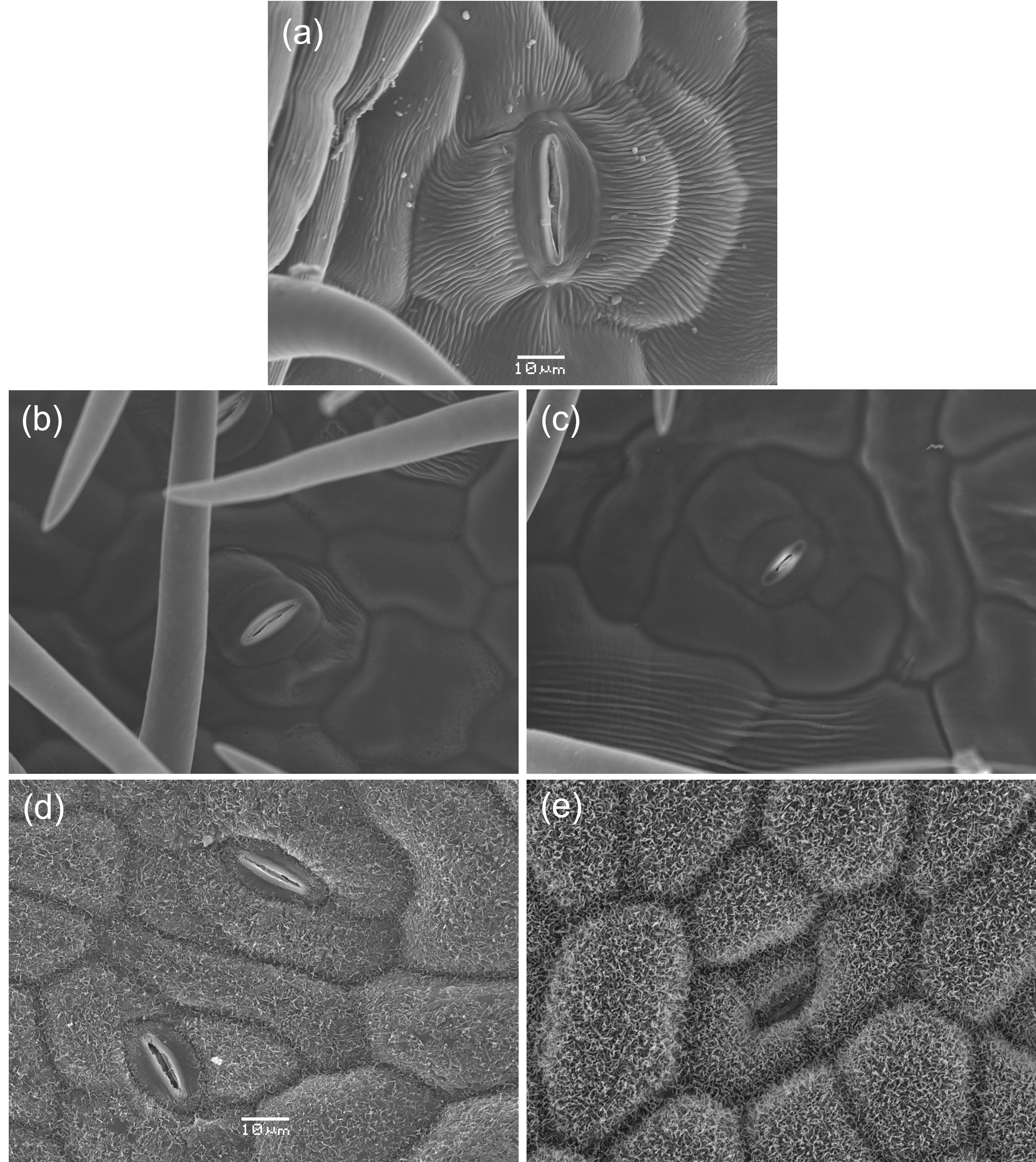

Supplement: mcae098_suppl_Supplementary_Figures [file mcae098_suppl_supplementary_figures.zip › aob-24221-s09.jpg]
