# Supplementary material for: Physiological and structural traits contribute to thermotolerance in wild Australian cotton species
Source: Ann Bot. 2024 Jul 9;135(3):577–88. doi: 10.1093/aob/mcae098 (PMC11897598; doi:10.1093/aob/mcae098)
Supplement: mcae098_suppl_Supplementary_Tables_S1-S15 [file mcae098_suppl_supplementary_tables_s1-s15.docx]

Table S1. ANOVA table for RGR.

| ANOVA table | SS | DF | MS | F (DFn, DFd) | P value |
| --- | --- | --- | --- | --- | --- |
| Interaction | 0.00259 | 4 | 0.000646 | F (4, 30) = 26.11 | P<0.0001 |
| Species | 0.02229 | 4 | 0.005573 | F (4, 30) = 225.1 | P<0.0001 |
| Temperature | 0.00017 | 1 | 0.000174 | F (1, 30) = 7.029 | P=0.0127 |
| Residual | 0.00074 | 30 | 2.48E-05 |  |  |

Table S2. ANOVA table for aboveground biomass.

| ANOVA table | SS | DF | MS | F (DFn, DFd) | P value |
| --- | --- | --- | --- | --- | --- |
| Interaction | 1.127 | 4 | 0.2817 | F (4, 30) = 45.02 | P<0.0001 |
| Row Factor | 1.506 | 4 | 0.3765 | F (4, 30) = 60.19 | P<0.0001 |
| Column Factor | 0.06609 | 1 | 0.06609 | F (1, 30) = 10.56 | P=0.0028 |
| Residual | 0.1877 | 30 | 0.006256 |  |  |

Table S3. ANOVA table for CO_2_ assimilation.

| ANOVA table | SS | DF | MS | F (DFn, DFd) | P value |
| --- | --- | --- | --- | --- | --- |
| Interaction | 817.2 | 4 | 204.3 | F (4, 30) = 124.4 | P<0.0001 |
| Species | 381.7 | 4 | 95.42 | F (4, 30) = 58.12 | P<0.0001 |
| Temperature | 35.46 | 1 | 35.46 | F (1, 30) = 21.60 | P<0.0001 |
| Residual | 49.25 | 30 | 1.642 |  |  |

Table S4. ANOVA table for *ETR*.

| ANOVA table | SS | DF | MS | F (DFn, DFd) | P value |
| --- | --- | --- | --- | --- | --- |
| Interaction | 2795 | 4 | 698.8 | F (4, 30) = 2.372 | P=0.0747 |
| Species | 14678 | 4 | 3669 | F (4, 30) = 12.45 | P<0.0001 |
| Temperature | 24069 | 1 | 24069 | F (1, 30) = 81.69 | P<0.0001 |
| Residual | 8839 | 30 | 294.6 |  |  |

Table S5. ANOVA table for *CE*.

| ANOVA table | SS | DF | MS | F (DFn, DFd) | P value |
| --- | --- | --- | --- | --- | --- |
| Interaction | 0.01045 | 4 | 0.002612 | F (4, 30) = 71.80 | P<0.0001 |
| Species | 0.01055 | 4 | 0.002637 | F (4, 30) = 72.47 | P<0.0001 |
| Temperature | 0.0006107 | 1 | 0.0006107 | F (1, 30) = 16.79 | P=0.0003 |
| Residual | 0.001092 | 30 | 0.00003638 |  |  |

Table S6. ANOVA table for *E*.

| ANOVA table | SS | DF | MS | F (DFn, DFd) | P value |
| --- | --- | --- | --- | --- | --- |
| Interaction | 0.0003992 | 4 | 0.0000998 | F (4, 30) = 119.2 | P<0.0001 |
| Species | 7.727E-05 | 4 | 0.00001932 | F (4, 30) = 23.08 | P<0.0001 |
| Temperature | 0.0002586 | 1 | 0.0002586 | F (1, 30) = 309.0 | P<0.0001 |
| Residual | 2.511E-05 | 30 | 8.369E-07 |  |  |

Table S7. ANOVA table for leaf temperature.

| ANOVA table | SS | DF | MS | F (DFn, DFd) | P value |
| --- | --- | --- | --- | --- | --- |
| Interaction | 17.9 | 4 | 4.475 | F (4, 30) = 26.48 | P<0.0001 |
| Species | 3.672 | 4 | 0.9179 | F (4, 30) = 5.431 | P=0.0021 |
| Temperature | 547.6 | 1 | 547.6 | F (1, 30) = 3240 | P<0.0001 |
| Residual | 5.071 | 30 | 0.169 |  |  |

Table S8. ANOVA table for leaf dissection ratio.

| ANOVA table | SS | DF | MS | F (DFn, DFd) | P value |
| --- | --- | --- | --- | --- | --- |
| Treatment (between columns) | 1.032 | 4 | 0.2579 | F (4, 15) = 77.15 | P<0.0001 |
| Residual (within columns) | 0.05014 | 15 | 0.003343 |  |  |
| Total | 1.082 | 19 |  |  |  |

Table S9. ANOVA table for average individual leaf area.

| ANOVA table | SS | DF | MS | F (DFn, DFd) | P value |
| --- | --- | --- | --- | --- | --- |
| Interaction | 111.1 | 4 | 27.78 | F (4, 39) = 15.39 | P<0.0001 |
| Species | 250 | 4 | 62.51 | F (4, 39) = 34.64 | P<0.0001 |
| Temperature | 1.971 | 1 | 1.971 | F (1, 39) = 1.092 | P=0.3025 |
| Residual | 70.39 | 39 | 1.805 |  |  |

Table S10. ANOVA table for reflectance at 0°.

| ANOVA table | SS | DF | MS | F (DFn, DFd) | P value |
| --- | --- | --- | --- | --- | --- |
| Treatment (between columns) | 1.232 | 4 | 0.308 | F (4, 25) = 9.106 | P=0.0001 |
| Residual (within columns) | 0.8457 | 25 | 0.034 |  |  |
| Total | 2.078 | 29 |  |  |  |

Table S11. ANOVA table for reflectance at 10°.

| ANOVA table | SS | DF | MS | F (DFn, DFd) | P value |
| --- | --- | --- | --- | --- | --- |
| Treatment (between columns) | 10.45 | 4 | 2.614 | F (4, 25) = 20.28 | P<0.0001 |
| Residual (within columns) | 3.222 | 25 | 0.129 |  |  |
| Total | 13.68 | 29 |  |  |  |

Table S12. ANOVA table for total leaf area.

| ANOVA table | SS | DF | MS | F (DFn, DFd) | P value |
| --- | --- | --- | --- | --- | --- |
| Interaction | 0.4175 | 4 | 0.104 | F (4, 30) = 41.91 | P<0.0001 |
| Species | 1.068 | 4 | 0.267 | F (4, 30) = 107.3 | P<0.0001 |
| Temperature | 0.001938 | 1 | 0.002 | F (1, 30) = 0.7780 | P=0.3848 |
| Residual | 0.07472 | 30 | 0.002 |  |  |

Table S13. ANOVA table for leaf area *RGR*.

| ANOVA table | SS | DF | MS | F (DFn, DFd) | P value |
| --- | --- | --- | --- | --- | --- |
| Interaction | 0.005773 | 4 | 0.001 | F (4, 39) = 6.407 | P=0.0005 |
| Species | 0.008064 | 4 | 0.002 | F (4, 39) = 8.949 | P<0.0001 |
| Temperature | 0.0002001 | 1 | 2E-04 | F (1, 39) = 0.8881 | P=0.3518 |
| Residual | 0.008786 | 39 | 2E-04 |  |  |

Table S14. ANOVA table for *SLA*.

| ANOVA table | SS | DF | MS | F (DFn, DFd) | P value |
| --- | --- | --- | --- | --- | --- |
| Interaction | 5636 | 4 | 1409 | F (4, 30) = 6.939 | P=0.0004 |
| Species | 18973 | 4 | 4743 | F (4, 30) = 23.36 | P<0.0001 |
| Temperature | 1923 | 1 | 1923 | F (1, 30) = 9.471 | P=0.0044 |
| Residual | 6092 | 30 | 203.1 |  |  |

Table S15. ANOVA table for stomatal conductance to water (*g_sw_*).

| ANOVA table | SS | DF | MS | F (DFn, DFd) | P value |
| --- | --- | --- | --- | --- | --- |
| Interaction | 0.991 | 4 | 0.248 | F (4, 30) = 328.6 | P<0.0001 |
| Species | 0.039 | 4 | 0.01 | F (4, 30) = 12.78 | P<0.0001 |
| Temperature | 0.028 | 1 | 0.028 | F (1, 30) = 37.54 | P<0.0001 |
| Residual | 0.023 | 30 | 8E-04 |  |  |
